# Microflow Mechanism of Oil Displacement by Viscoelastic Hydrophobically Associating Water-Soluble Polymers in Enhanced Oil Recovery

**DOI:** 10.3390/polym10060628

**Published:** 2018-06-07

**Authors:** Huiying Zhong, Yuanyuan Li, Weidong Zhang, Hongjun Yin, Jun Lu, Daizong Guo

**Affiliations:** 1 Key Laboratory for Enhanced Oil & Gas Recovery of the Ministry of Education, Northeast Petroleum University, Daqing 163318, China; 18545070325@163.com (Y.L.); zwdhenan@163.com (W.Z.); nepuwzh@163.com (D.G.); 2 McDougall School of Petroleum Engineering, University of Tulsa, Tulsa, OK 74104, USA; jun-lu@utulsa.edu

**Keywords:** hydrophobically associating water-soluble polymers, viscoelasticity, shear-thickening, two-phase flow, rheology, microflow mechanism

## Abstract

Polymer flooding plays an important role in enhanced oil recovery (EOR), particularly in China, where partially hydrolyzed polyacrylamide (HPAM) and hydrophobically associating water-soluble polymers (HAWP) are used in onshore and offshore reservoirs, respectively. Many researchers have highlighted the elasticity of HPAM, which can be used to improve the sweep efficiency, i.e., the ratio of the area swept by an injected fluid to the oil area. On the other hand, fewer studies exist on the elasticity of HAWP. In this study, we investigate the flow of HAWP and Xanthan solutions with identical viscosities in core experiments in terms of elasticity; results reveal that the HAWP can produce shear thickening in the core. The constitutive equation for the HAWP can be obtained using the simulation results matched with the experimental data. On the basis of these experiments, we established a two-phase flow model of a polymer and oil, including the continuity, momentum, constitutive, and phase equations. The volume-of-fluid (VOF) method was used to track the interface between the two phases. A complex pore model was established based on the glass-etched model used in the experiment. We used the OpenFOAM platform to solve the mathematical model. The saturation, pressure, and stress tensor distributions were obtained. The results show that the displacement efficiency increased as the elasticity of the polymer increased; accordingly, the elasticity can enlarge the sweep area and decrease the residual oil saturation. As the elasticity increases, the stresses (the first normal stress, second normal stress, and shear stress) increase. Finally, the results obtained in this study can be used as a guideline in polymer design, screening, and optimization in the polymer flooding oilfields.

## 1. Introduction

**Figure 1:**
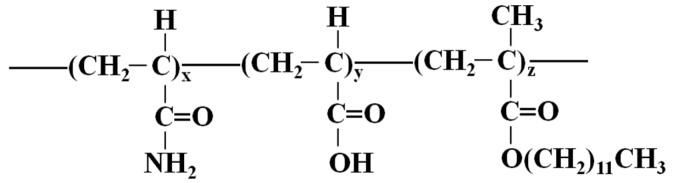
Molecular structure of HAWP: x = 30–100, y = 0–50, and z = 0.01–1.

**Figure 2:**
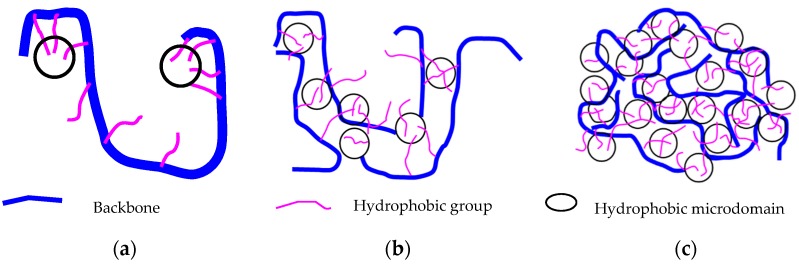
Schematic diagram of HAWP association (*C*: concentration; C*: critical concentration): (**a**) *C* < C*, intramolecular association; (**b**) *C* < C*, intermolecular association; and (**c**) *C* > C*, the formation of a network structure by the association of molecules.

In a world of growing energy demands, although shale oil, coal, natural gas and other new energies continue to develop, oil remains the leading source of primary energy [1,2,3,4]. Global oil consumption increased from 0.8 million barrels per day in 2000 to approximately 100 million barrels of consumption per day in 2017. However, the increase in production has been slow, comprising only half that of consumption. In particular, the recent low oil prices have forced oil companies to enhance their oil recovery as a response to price challenges. Therefore, chemical flooding EOR techniques, including alkali/surfactant/polymer (ASP) flooding, foam flooding, and CO_2_ flooding, are being increasingly applied [5,6,7]. However, polymer flooding plays the most important role, particularly in the Chinese petroleum industry. In Daqing, the largest oilfield in China, polymer flooding has entered the commercial stages. The oil recovery can be increased 13% in comparison to that of water flooding [8]. The application of polymer flooding can guarantee continued supply in the future. Therefore, researchers have focused on the mechanisms of polymer flooding. In the early stage, a polymer can enhance oil recovery, increasing the viscosity and reducing water permeability, which can, accordingly, change the flow mobility ratio [9,10,11]. There are many such types of polymers, including synthetic polymers and biopolymers. To date, the Daqing oilfield has predominantly applied a synthetic polymer of HPAM. This material has specific advantages, such as the ability to tolerate high mechanical forces, a low cost, and an overall resistance to bacterial attack [10,12]. However, it is sensitive to brine salinity, making it ineffective for offshore oil due to the high salinity of sea water. The other group of polymers is the HAWP, which, in comparison to other polymers, have a smaller number of hydrophobic groups incorporated into the polymer backbone. In China, this polymer has been used in the BH offshore heavy oilfield. The high salinity and hardness of the water as well as the high viscosity of the oil require the polymer to be both salt-resistant and have good viscosity. Additionally, the polymer should have a strong anti-mechanical degradation ability as a result of the completion involving wire-wrapped and screen gravel-packed sand. Moreover, the long-term stability of the displacing fluid in the formation is important because of the large well spacing in offshore fields [13,14,15]. Therefore, HAWP can overcome the above shortcomings to satisfy these requirements. Its molecular structure is shown in Figure 1 [16]. HAWP is designed and synthesized as a small portion of a hydrophobic comonomer, which is to be incorporated into the backbone of a polymer. There are two general methods to incorporate hydrophobic moieties into water-soluble polymer chains: direct copolymerization of hydrophobic and water-soluble monomers and post-polymerization functionalization [17]. The former is the most common method of achieving associating polymer synthesis. Hydrophobically associating block copolymers are prepared by anionic polymerization [18,19,20]. Associating polymers involve acrylamide and free radical polymerization. Although other monomers have been used, arylamide is the most successful and most suited for producing high molecular weight water-soluble polymers [21,22]. This polymer is prepared by incorporating hydrophobic groups into the polymer after the polymerization process. It has potential for use in mobility control; its advantages include its constant viscosity when temperatures increase, attributable to its interchain association. It also can tolerate high salinities [23,24]. The viscosity can increase rapidly when the concentration is larger than the critical association concentration because of the network structure formed by the association of molecules, as shown in Figure 2 [25]. HAWP have been tested to be shear-stable at high flow rates. They showed a higher resistance and overall higher residual resistance factors than conventional polyacrylamides; moreover, they did not exhibit any injection problems. Therefore, they are applied in Chinese offshore reservoirs.

In many studies, researchers have predominantly concentrated on HAWP characteristics such as its molecular structure, rheology, field applications, and enhanced oil recovery [26,27,28,29,30,31]. However, fewer studies exist on the elasticity of HAWP flow in porous media. The elasticity of HPAM has attracted the attention of researchers for many years. When a polymer solution flows in a capillary tube with a constant diameter, the elasticity cannot be observed. However, in porous media, the shape and diameter of the pores and throat change rapidly, so elasticity can be obviously observed. Many investigators have studied the flow of polymer through porous media. The total pressure drop of polymer flooding is separated into the contributions of two parts, viscous contribution and elastic contribution parts; elastic parts can also enhance the oil recovery. In a 2000 study involving the HPAM and pure, viscous Xanthan solution with the same viscosity displacement oil on glass etched and core respectively, the results showed that the elasticity could enhance oil recovery and increase microsweep efficiency [32]. Xia et al. observed that, at higher elasticity, the residual oil could be mobilized because of the higher microforces that act on the different types of oil droplets in the pores, causing the oil droplet to change its shape and move. In the micro glass-etched experiment, they observed that the oil in the deadend was “pulled out” and “tripped” because of the elasticity effect [33]. Chen and Bogue proposed a model that can be used to calculate the residual oil saturation by a first normal stress difference as well as capillary number [34]. The first normal stress difference can present the elasticity of the polymer; results indicated a decrease in the residual oil saturation as the first normal stress difference increased. With the development of computers and computational methods, computational fluid dynamics (CFD) was used to study the elastic effect on the polymer displacement oil. In 2006, Yin et al. showed that the residual oil remained in different forms and that elasticity could make the residual oil move; concurrently, the study indicated that the microsweep area enlarged as elasticity increased [35]. In 2012, Yin et al. concluded from simulation results that viscoelasticity was the main property of a polymer in the increase of the microscopic sweep efficiency; the study also concluded that, with a higher Reynolds number, the microscale sweep efficiency was improved [36]. In 2013, Qi et al. simulated the elastic polymer flow in complex pores using the finite element method [37]. The pore model was derived from the high-resolution micro-CT images of a typical sandstone. The results showed that the fluid more deeply invaded the dead pores with higher elasticity; for higher Reynolds numbers, the fluid further penetrated the dead pores.

In this study, we investigate the HAWP rheology in porous media by comparing elastic HAWP and pure viscous Xanthan flow in cores; the aim of this experiment is to investigate the elasticity of the HAWP effect in porous media. On the basis of the experiment, the constitutive equation describing the rheology of HAWP is confirmed. In addition, CFD simulations were used to simulate the viscoelastic polymer displacement oil in the complex pore model. The results are significant for the optimum design of HAWP solutions in chemical flooding EOR processes.

## 2. Rheological Characterization of the Flow of HAWP Solutions in Porous Media

The important characteristics of a polymer solution flowing in porous media is that, when the flow velocity is low, it shows shear-thinning; shear-thickening appears when the flow velocity is high [38,39]. When single-phase flow is observed in the core, the relation of flow rate, shear rate, pressure gradient, and apparent viscosity can be expressed by the power-law Darcy Equation. The shear rate and apparent viscosity were calculated by Morais [40] in 2009 using the non-Newtonian law; they are expressed as:

(1) $$\dot{\gamma }=\alpha \frac{Q}{A\sqrt{K\phi }}$$

(2) $$\eta \left(\dot{\gamma }\right)=-\frac{AK\nabla P}{Q}$$

where $\dot{\gamma }$ is the apparent shear rate, 1/s; *α* is the non-Newtonian correction for a power-law fluid, in this paper, it is 4; *Q* is the flow rate, m^3^/s; *A* is the cross-sectional area of the core, m^2^; *ϕ* is the porosity; *K* is the permeability, m^2^; $\eta \left(\dot{\gamma }\right)$ is the apparent viscosity, Pa·s.

### 2.1. Experimental Solutions

In this study, APP-4, a type of HAWP synthesized by acrylamide and anionic copolymer monomer with a hydrophobic monomer unit, was employed. The molecular weight of the HAWP solutions used was 1.1 × 10^7^ Da and varied with different concentration; the experimental water was prepared using double-distilled water, and its salinity is 9947.8 mg/L, which is the same as that of the BH oilfield actual formation water. The concentrations of the HAWP solutions are 1500 mg/L, 1750 mg/L, and 2000 mg/L. In this study, the other type of solution used was Xanthan, a pure viscous fluid, and a solution of concentration 3000 mg/L was prepared.

### 2.2. Core Material and Initialization

The core of this experiment was made using a quartz sand epoxy resin of diameter 25 mm and length 80 mm. The average permeability and porosity were 2000 × 10^−3^ µm^2^ and 25~30%, respectively. The characterizations were based on the properties of the BH oilfield reservoir.

### 2.3. Measurement Conditions

**Figure 3:**
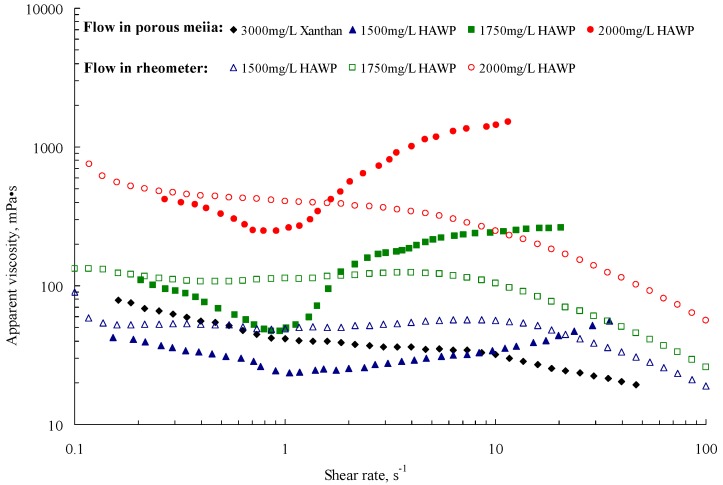
Apparent viscosity vs. shear rate for different polymer solutions.

Measurements were made at 65 °C, the same temperature as the reservoir temperature. The flow rate of the pump was 0.003~10 mL/min and was increased from low to high; when the flow rate was constant, the pressure difference between the inlet and outlet was recorded. Figure 3 shows the diagram of apparent viscosity versus shear rate in the core.

In Figure 3, the hollow circle represents the results of the measurements made when HAWP solutions were flowing in the rheometer. We can observe that the viscosity decreased with increasing shear rate. This indicates the shear-thinning characteristic. However, when the single HAWP flowed in the porous media, the apparent viscosity decreased as the shear rate increase when the shear rate was low; correspondingly, it increased with shear rate increase when the shear rate was high. Therefore, the HWAP exhibited the shear-thickening phenomenon when they flowed in the core. The main reason for this occurrence is that shear effect only occurred when a fluid was flowing in the rheometer; however, when a fluid flowed in the core, elasticity was obvious because of the change in the shape and size of the pore and throat. Therefore, stretching can cause the polymer molecule to associate and the apparent viscosity to increase. Moreover, we observed that the Xanthan solution without elasticity did not exhibit the shear-thickening phenomenon in the core. This can validate that the reason mentioned above is correct. As shown in this Figure, we also determine the position where the rheology changes from shear-thinning to shear-thickening to maintain immobilization for different HAWP concentrations. It was observed that the position where shear-thickening occurred was not related to the concentration of the polymer; instead, it was potentially related to the molecular weight and structure.

**Figure 4:**
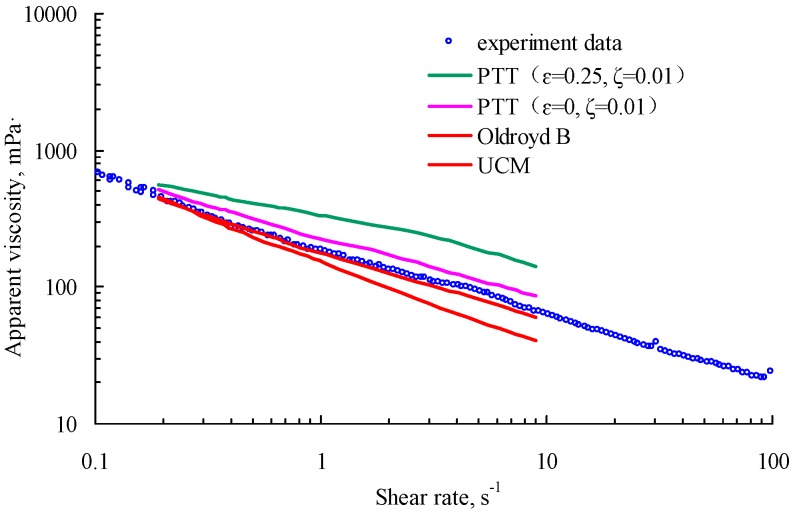
Apparent viscosity matching with shear rate diagram.

Different rheologies can be shown for different polymers. To describe the rheology accurately in numerical simulations, we matched the apparent viscosity versus shear rate curve obtained for the numerical and experimental results. In Figure 4, the circle represents the point where the results were measured with a rheometer for 1750 mg/L HAWP solutions, and the different colored lines are the results of numerical simulation using different constitutive equations. The simulations were performed using a rectangular model, the ratio of which, length to width, was large enough and the flow was two-dimensional, incompressible, and isothermal. As shown in Figure 4, the deviation in the Oldroyd-B model results was minimum. Therefore, in the following numerical simulation, we used Oldroyd-B as the constitutive equation.

## 3. Mathematical Model

In this study, the mathematical model includes the continuity, momentum, and constitutive equations. Because this study included two phases, the polymer and oil, interface tracking was an important question; accordingly, an interface tracking equation was necessary. In this study, we adopted the VOF method.

### 3.1. Continuity Equation

This problem includes two continuity equations that can be written uniformly as one equation:

(3) $$\frac{\partial \rho }{\partial t}+\nabla \cdot \left(\rho U\right)=0$$

where *U* is the velocity vector, m/s, which can be resolved into components of the velocity in the *x* and *y* directions. *ρ* is the density, when the point considered is in the polymer solution, it represents the density of the polymer solution. Otherwise, it represents the density of oil. In a two-phase transition zone, it is the mixture density; *t* is the flow time, s.

### 3.2. Momentum Equation

If it is a single-phase flow, the momentum equation is represented as:

(4) $$\frac{\partial \left(\rho U\right)}{\partial t}+\nabla \cdot \left(\rho UU\right)=-\nabla p+\nabla \cdot \tau$$

In this equation, *τ* includes the viscoelastic polymer contribution *τ*_s_, given by *τ*_s_ = 2*η*_s_*D*, and the solvent contribution *τ*_p_, which can be calculated by the constitutive equation. However, in two-phase flow problems, interfacial tension will induce a pressure jump. If the fluids are in equilibrium, *f*_σ_ is the external body-force that is mechanically balanced by a pressure jump across the interface. Therefore, the momentum equation is rewritten as:

(5) $$\frac{\partial \left(\rho U\right)}{\partial t}+\nabla \cdot \left(\rho UU\right)-\left(\eta_{s}+\eta_{p}\right)\nabla \cdot \left(\nabla U\right)=-\nabla p+\nabla \cdot \tau_{p}-\eta_{p}\nabla \cdot \left(\nabla U\right)+f_{\sigma }$$

where *η*_s_ is the viscosity of the solvent and *η*_p_ is the viscosity of the polymer solutions. Then, *f*_σ_ can be expressed as:

(6) $$f_{\sigma }=\nabla p=-\sigma \left(\nabla \cdot \left(\frac{\nabla \alpha }{\left|\nabla \alpha \right|}\right)\right)\left(\nabla \alpha \right)$$

where the fluid volume fraction *α* is defined as *α =* 1 for a point inside the polymer and *α =* 0 for a point inside the oil. Therefore, the density and dynamic viscosity in the equations of momentum are:

(7) $$\rho =\alpha \rho_{p}+\left(1-\alpha \right)\rho_{o},\;\mu =\alpha \mu_{p}+\left(1-\alpha \right)\mu_{o}$$

where *ρ*_p_ is the polymer density and *ρ*_o_ is the oil density. They are substituted into the continuity equation, which can be reformulated in this manner:

(8) $$\frac{\partial \alpha }{\partial t}+U\cdot \nabla \alpha =0$$

The equation system is closed with the constitutive equations of the two phases. The polymer rheology is described by the Oldroyd-B Equation, which is written as:

(9) $$\tau_{p}+\lambda \overset{\nabla }{\tau_{p}}=\eta_{0}D$$

The power-law equation is used to describe the oil rheology:

(10) $$\eta_{o}=K{\dot{\gamma }}^{n}$$

where: *η*_0_ is the zero shear viscosity of the polymer solutions, Pa·s, and *K* is the consistency coefficient, Pa·s^n+1^. $\dot{\gamma }$ is the shear rate, s^−1^. These equations complete the mathematical description of the two-fluid system for laminar flow.

## 4. Physical Model

**Figure 5:**
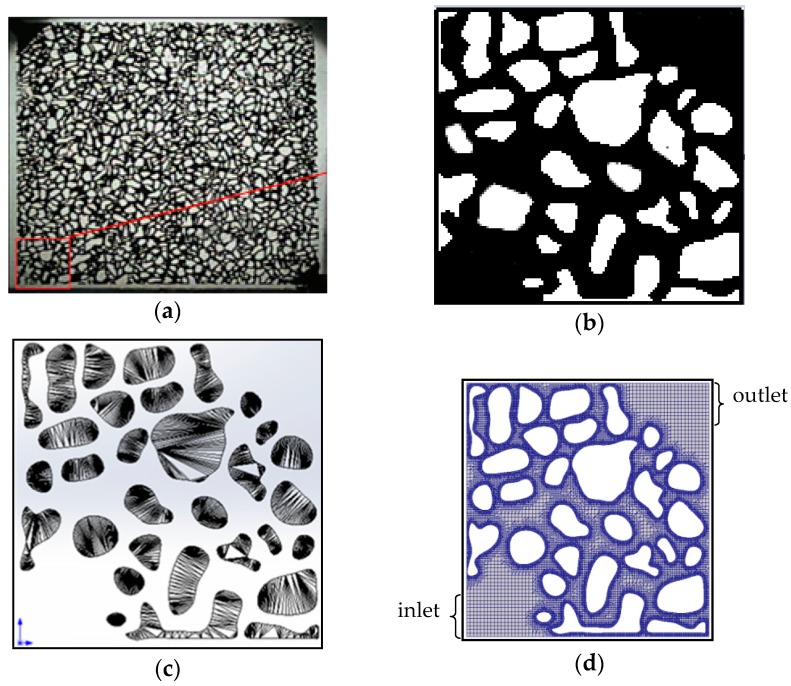
Mesh generation process for the physical simulation model: (**a**) the glass-etched model used in the microexperiment; (**b**) the black and white digitized model; (**c**) STL skeleton grid generated by solidworks; and (**d**) pore meshes generated by OpenFOAM.

In this study, the physical model used was based on the glass etched in the experiments, shown in Figure 5a. In consideration of the memory and computation time of the computer, we picked one portion from the whole physical model: the red portion, as shown in Figure 5b–d, which represents the solid skeleton grid model and pore mesh subdivided by the snappyHexMesh solver in OpenFOAM. (OpenCFD Ltd., Bracknell, UK) There were 71,613 grids; the maximum radius of the pore was 1.24 mm and the minimum radius was 0.08 mm. This model can reflect the topology and complexity of the real porous media. Elasticity can be obviously observed in this complex model.

## 5. Numerical Simulation

In this study, Equations (3) and (5)–(8) constitute the entire governing system of equations. They are discrete and the coupling of velocity and pressure has been addressed. The solution procedure includes: ① initialization of all the fields (*α*, *U*, *τ* and *p*), ② solving the Equation (6) so that a new *α* can be calculated, along with the density and viscosity, ③ solve the momentum Equation (5) so that the velocity field can be obtained. Then, on the basis of continuity Equation (3), the correction velocity and the new velocity field can be calculated. Here, the PISO (Pressure Implicit Split Operator) algorithm was used; ④ solve the constitutive Equation, so that the stress tensor can be calculated. Return to step ② until the target time is reached.

In this study, we investigated four displacement patterns: water flooding, no elastic polymer flooding, and two different elastic polymer solution floodings. The parameters are shown in Table 1.

### 5.1. Volume Fraction Characteristics

**Figure 6:**
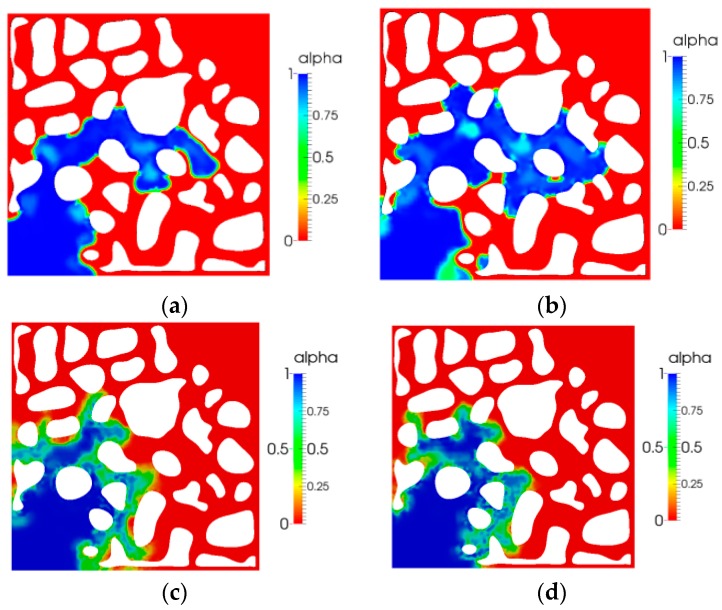
Volume fraction distribution (*t =* 11 s): (**a**) water flooding; (**b**) polymer flooding (no elasticity); (**c**) polymer flooding (*λ =* 0.01); and (**d**) polymer flooding (*λ =* 0.03).

In this study, the phase volume fraction can represent the saturation of water. Figure 6 shows the saturation for the four patterns at 11 s. As shown in Figure 6, in comparison with the case of no elastic polymer displacement, water movement was faster and the fingers grew faster in the water displacement oil. In comparison with the water flooding with no elastic polymer flooding, the elastic polymer flooding case reveals a steady front and better sweep area. As the relaxation time increased, the sweep area was larger.

**Figure 7:**
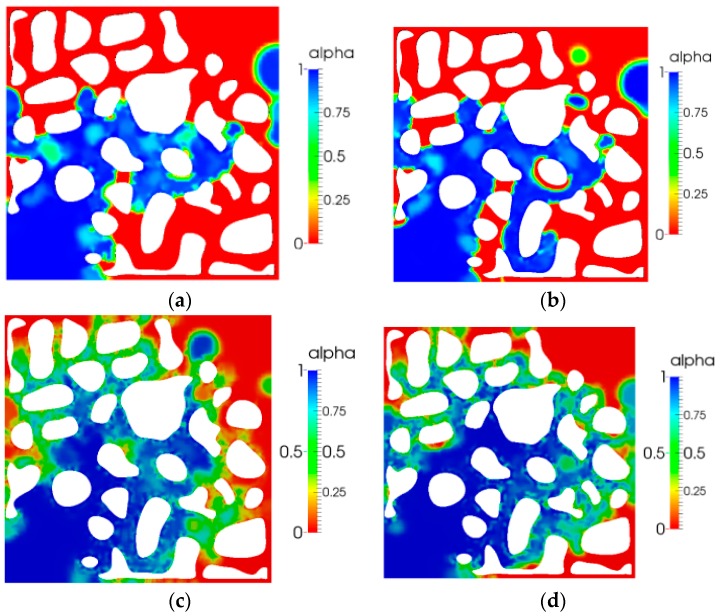
Displacement fluid breakthrough time: (**a**) 15 s water flooding; (**b**) 16 s no elastic polymer flooding; (**c**) 19 s viscoelastic polymer flooding (*λ =* 0.01); and (**d**) 21 s viscoelastic polymer flooding (*λ =* 0.03).

Figure 7 shows the breakthrough times of the four different patterns. It indicated that the breakthrough time of water flooding was earlier than that of no elastic polymer flooding. A comparison between the breakthrough time of no elastic polymer flooding with that of viscoelastic polymer flooding indicated that the breakthrough time of elastic polymer flooding was later. Moreover, as the elasticity increased, the front breakthrough time was later. A comparison of Figure 7c,d also indicates that the sweep area enlarged with an increase in relaxation time.

**Figure 8:**
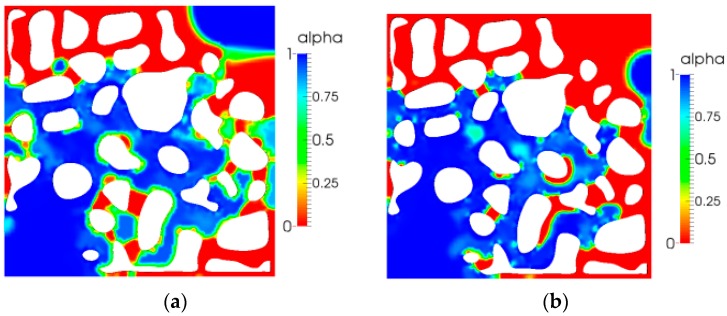

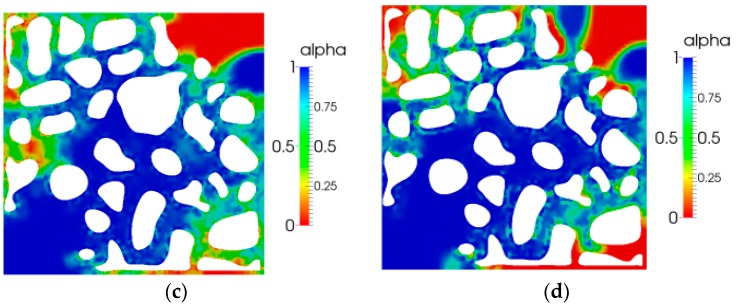
Saturation distribution after breakthrough (*t* = 28 s): (**a**) water flooding; (**b**) no elastic polymer flooding; (**c**) viscoelastic polymer flooding (λ = 0.01); and (**d**) viscoelastic polymer flooding (λ = 0.03).

Figure 8 shows the saturation distribution at *t* = 28 s after the displacement fluid breakthrough. Because of poor mobility ratio, the residual oil saturation of water flooding was the largest, and the residual oil saturation decreased as the elasticity of polymer increased, as shown in Figure 8c,d.

### 5.2. Pressure Distribution

**Figure 9:**
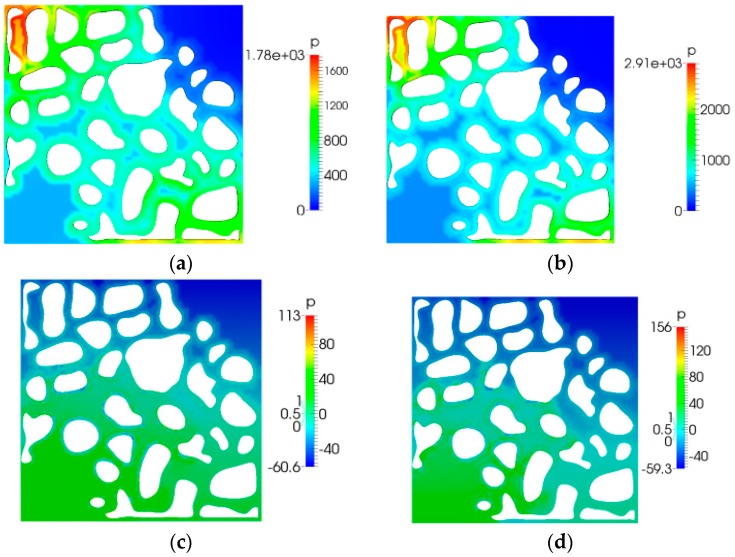
Volume fraction distribution (*t* = 11 s): (**a**) water flooding; (**b**) no elastic polymer flooding; (**c**) viscoelastic polymer flooding (λ = 0.01); and (**d**) viscoelastic polymer flooding (λ = 0.03).

**Figure 10:**
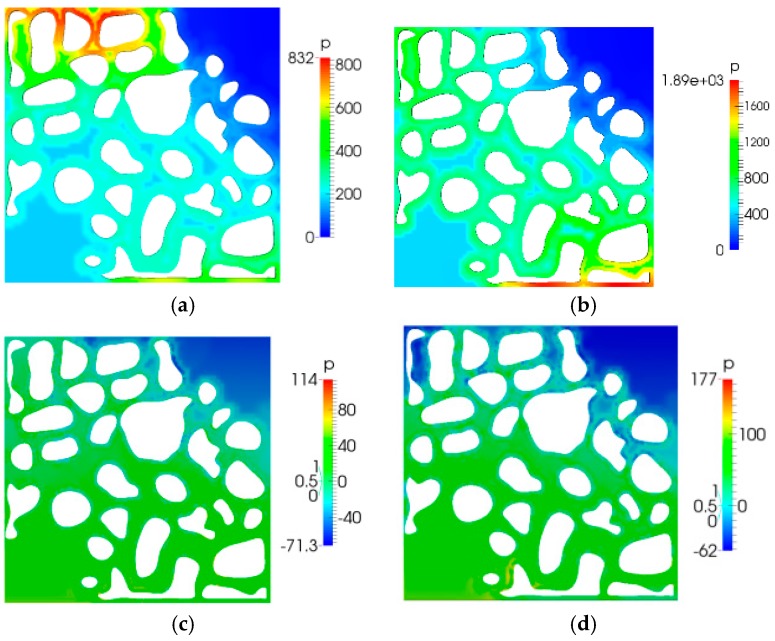
Displacement fluid front breakthrough times: (**a**) 15 s; (**b**) 16 s; (**c**) 19 s; and (**d**) 21 s.

Figure 9 shows the pressure distribution diagram. As shown in Figure 9, in comparison to the case of water flooding, when there was no elastic polymer flooding, an additional pressure drop was induced; moreover, the elastic polymer flooding could also produce an additional pressure drop compared to the case of viscous polymer flooding. As the elasticity increased, the additional pressure drop increased. Moreover, we observed that the pressure in elastic polymer flooding was distributed more uniformly in comparison to the cases of water flooding and no elastic polymer flooding. These two flooding techniques could both produce a local high pressure area. Figure 10 shows the pressure distribution when the displacement fluid front broke through. It is shown that the pressure variation is the same as that before the breakthrough.

**Figure 11:**
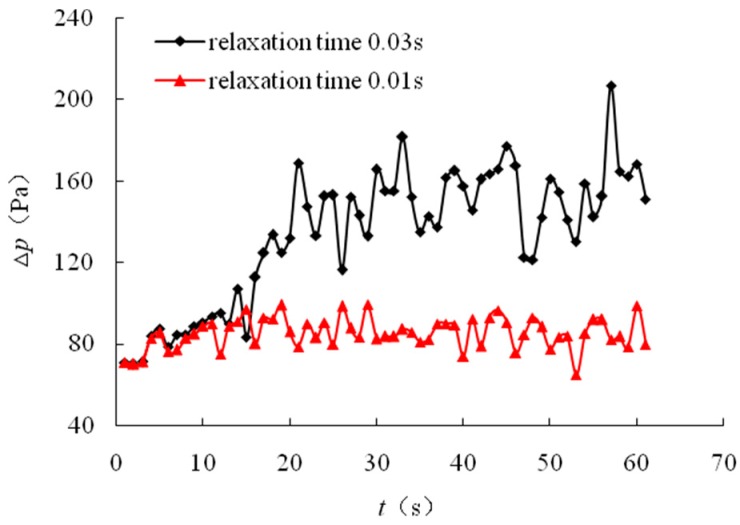
Displacement pressure drop vs. time for different elastic polymer solutions.

On the basis of the pressure distribution, the pressure drop between inlet and outlet versus time is plotted in Figure 11. As can be seen, the pressure drop increased until the displacement fluid broke in the outlet.

As shown in Figure 11, early in the displacement process, as a result of the same inflow rate and outlet conditions, their pressure drops were also identical. Subsequently, as the elasticity increased, the pressure drop increased rapidly. We also observed that, before the front breakthrough, the pressure drop increased rapidly; in contrast, after the breakthrough, the pressure increase slowed. We can conclude that the elasticity of the polymer can produce an additional pressure drop that can increase the sweep efficiency and enhance oil recovery.

### 5.3. Stress Tensor Distribution

**Figure 12:**
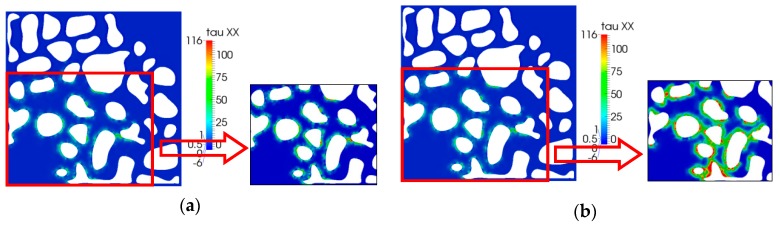
First normal stress (*τ*_xx_) distribution: (**a**) *λ =* 0.01 s; (**b**) *λ =* 0.05 s.

In this study, the problem is the two-dimensional flow, and the stress tensor included three components. Figure 12 is the first normal stress distribution for different relaxation times. As shown in this Figure, an obvious change in the first normal stress cannot be observed because of the low relaxation time. However, local enlargement (Figure to the right of the corresponding Figure) can reveal that the normal stress tensor increases as the relaxation time increases, especially in the thin pores and throat; additionally, the shape of the pore also changes sharply where the fluid becomes more elastic, suggesting elasticity can play an important role. This is the main reason that elasticity can contribute to enhanced oil recovery. This has been discussed in many reports, particularly those pertaining to the single-phase flow problem [41,42,43,44,45].

**Figure 13:**
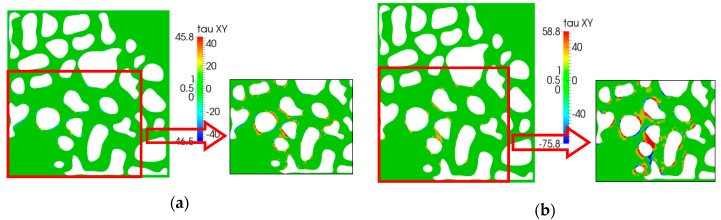
Shear stress (*τ*_xy_) distribution: (**a**) *λ =* 0.01 s; (**b**) *λ =* 0.05 s.

The shear stress simulation results are shown in Figure 13. We can easily see that, as the elasticity increased, relaxation time increased; this was a result of the direction of shear stress occurring in the flow direction. The maximum of the shear stress increased obviously, especially near the walls of the pore.

**Figure 14:**
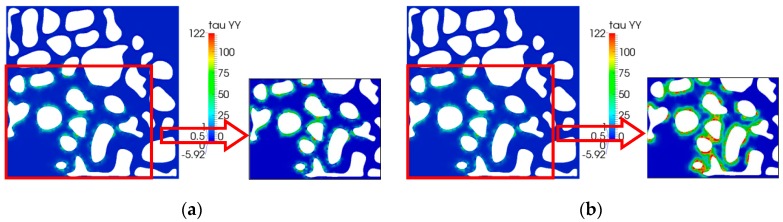
Shear stress (*τ*_xy_) distribution: (**a**) *λ =* 0.01 s; (**b**) *λ =* 0.05 s.

Figure 14 reveals the second normal stress distribution. As shown in the local enlargement Figure, the value was larger as the relaxation time increased. This phenomenon is in agreement with the results of the first normal stress mentioned above. They both validate that the elasticity can increase the displacement efficiency.

## 6. Conclusions

In this work, we focused on the mechanism of oil displacement using viscoelastic hydrophobically associating water-soluble polymers. The polymer flowing in porous media experiments reveals that the hydrophobically associating water-soluble polymer can exhibit shear thickening because of its elasticity; however, the viscous Xanthan solution only exhibited shear thinning. We observed that the position of occurrence was the same for different concentrations.

Furthermore, we established the mathematical model for solving the two-phase flow in porous media, which was developed for etched glass and can satisfy the geometry and topology of porous media.

We demonstrated that the polymer solution can have a stable front and better sweep area in comparison to water flooding. Elasticity is helpful in enlarging the sweep area. Before front breakthrough, the pressure drop between the inlet and outlet increased rapidly; subsequently, the increase was slow. As the elasticity increased, the additional pressure increased. As the elasticity increased, the first normal stress and shear stress both increased, especially in the small pore and throat.

## Figures and Tables

**Table 1:** Parameters of the four computational cases considered in this study.

Case	Flow Rate [m^3^/s]	Oil		Displacement Fluid				Interfacial Tension [mN/m]
		Density [kg/m^3^]	Viscosity [mPa·s]	Density [kg/m^3^]	Viscosity [mPa·s]		Relaxation Time [s]	
Waterflooding	1 × 10^−10^	860	9	1000	1		-	4.8
No elastic polymer flooding	1 × 10^−10^	860	9	900	12		-	4.8
Elastic polymer flooding (*λ* = 0.01)	1 × 10^−10^	860	9	900	*η*_s_	*η*_p_	0.01	4.8
					1	11		
Elastic polymer flooding (*λ* = 0.03)	1 × 10^−10^	860	9	900	1	11	0.03	4.8
